# Relative Invasion Risk for Plankton across Marine and Freshwater Systems: Examining Efficacy of Proposed International Ballast Water Discharge Standards

**DOI:** 10.1371/journal.pone.0118267

**Published:** 2015-03-12

**Authors:** Oscar Casas-Monroy, Robert D. Linley, Jennifer K. Adams, Farrah T. Chan, D. Andrew R. Drake, Sarah A. Bailey

**Affiliations:** 1 Great Lakes Laboratory for Fisheries and Aquatic Sciences, Fisheries and Oceans Canada, 867 Lakeshore Road, Burlington, ON, L7S 1A1, Canada; 2 Great Lakes Institute for Environmental Research, University of Windsor, 401 Sunset Ave., Windsor, ON N9B 3P4, Canada; 3 Department of Biological Sciences, University of Toronto Scarborough, 1265 Military Trail, Toronto, ON, M1C 1A4, Canada; Texas A&M University, UNITED STATES

## Abstract

Understanding the implications of different management strategies is necessary to identify best conservation trajectories for ecosystems exposed to anthropogenic stressors. For example, science-based risk assessments at large scales are needed to understand efficacy of different vector management approaches aimed at preventing biological invasions associated with commercial shipping. We conducted a landscape-scale analysis to examine the relative invasion risk of ballast water discharges among different shipping pathways (e.g., Transoceanic, Coastal or Domestic), ecosystems (e.g., freshwater, brackish and marine), and timescales (annual and per discharge event) under current and future management regimes. The arrival and survival potential of nonindigenous species (NIS) was estimated based on directional shipping networks and their associated propagule pressure, environmental similarity between donor-recipient ecosystems (based on salinity and temperature), and effects of current and future management strategies (i.e., ballast water exchange and treatment to meet proposed international biological discharge standards). Our findings show that current requirements for ballast water exchange effectively reduce invasion risk to freshwater ecosystems but are less protective of marine ecosystems because of greater environmental mismatch between source (oceanic) and recipient (freshwater) ecoregions. Future requirements for ballast water treatment are expected to reduce risk of zooplankton NIS introductions across ecosystem types but are expected to be less effective in reducing risk of phytoplankton NIS. This large-scale risk assessment across heterogeneous ecosystems represents a major step towards understanding the likelihood of invasion in relation to shipping networks, the relative efficacy of different invasion management regimes and seizing opportunities to reduce the ecological and economic implications of biological invasions.

## Introduction

Aquatic invasive species are nonindigenous organisms that establish and spread in a new environment, frequently resulting in negative impacts on biodiversity [1], ecological structure and function [2], and imperilment of fauna [3]. As the severity of impacts is influenced by multiple interacting drivers that are difficult to predict, prevention through vector management has often been cited as the best conservation strategy [4, 5]. Despite these recommendations, little attention has been directed at understanding efficacy of different vector management approaches, particularly at landscape scales, impeding the progress of science-based regulation and precluding assessments evaluating current and future ecological risk.

The unintentional release of nonindigenous species (NIS) in ballast water moved by transoceanic ships has long been recognized as a primary vector of aquatic invasions [6–9]. As a result, many countries have enacted ballast water management at landscape scales (i.e., at the scale of global or national shipping networks; [10]) to reduce the risk of species invasion and concomitant ecological change. Currently, the main requirement entails ballast water exchange (BWE), where vessels replace ballast water loaded at a foreign port with offshore oceanic water in an effort to reduce the abundance and diversity of coastal plankton transported between ports, thereby decreasing the likelihood of invasion because the number of individuals discharged in ballast water (propagule pressure) is an important determinant of invasion success [11].

The efficacy of BWE as a management strategy, however, is highly variable. Empirical studies indicate that BWE purges 70–100% of planktonic organisms entrained at the source port depending on ship type, method and location of exchange, and type of organism [12–16]. Ballast water exchange is considered most protective for freshwater recipient ports due to osmotic shock induced by the alternation of marine and fresh waters [17], and less effective for vessels conducting coastal exchange (50–200 nautical miles offshore) in comparison to mid-ocean exchange (> 200 nautical miles offshore) since plankton densities can be high in nearshore waters [15, 18, 19]. Furthermore, BWE is not a viable option for intra-regional vessels that remain within 50 nautical miles of shore; however, few studies have examined the ecological implications of BWE across landscape scales and global shipping networks involving many permutations of taxa, propagule pressure, and ecological conditions of source and recipient locations.

Due to concerns about limited ecological protection afforded by BWE, pending ratification of an international convention, vessels will soon be required to manage ballast water to meet numeric discharge standards limiting the density of organisms released to recipient ecosystems (the D-2 standards; [20]). It is expected that most vessels will install on-board multi-stage treatment systems such as filtration + electro-chlorination [21, 22], which are expected to physically remove large particles and chemically inactivate small particles in ballast water to meet the D-2 standards: less than 10 viable organisms ≥ 50 μm in minimum dimension per m^3^ (nominally ‘zooplankton’) and less than 10 viable organisms ≥ 10 μm and < 50 μm in minimum dimension per mL (nominally ‘phytoplankton’) [20]. Since plankton densities can be highly variable after BWE, in theory, treatment should offer risk reduction by consistently reducing plankton density to low levels. In addition, treatment is considered a safer and more broadly applicable management tool for transoceanic, coastal and domestic vessels. Concerns have been raised, however, that the D-2 standards are not stringent enough, as a proportion of ships can meet the standards without any ballast water management [10]. Further, concerns about the loss of the salinity barrier imposed by BWE have prompted Canadian and American regulators to propose enhanced requirements (BWE plus treatment) for vessels arriving to fresh water.

In general, the probability that a particular species will become established decreases as propagule pressure decreases [23–25], however, invasions are a stochastic process and the shape of the propagule pressure-establishment relationship is highly context-dependent [11, 26, 27]. The relationship is further complicated by the wide range of possible combinations of the number of individuals released per event, the frequency of release events, and the specific physical-chemical requirements and ecological interactions between arriving propagules and the recipient community [28–30]. As a result, it has not been possible to allocate a numeric invasion probability with confidence for specific inoculum densities. Even though the relative importance of different combinations has not been quantified, it has been postulated that invasion success is greater for multiple release events over time and space than for single, high-density releases, since repeated introductions may allow founding populations to overcome stochastic demographic and environmental limitations [11, 24, 31].

Using a mechanistic approach, the objective of this study is to evaluate the relative invasion risk to diverse ecosystems at landscape scales (i.e., across different directional shipping networks arriving to freshwater, brackish, and marine ports in Canada and the Laurentian Great Lakes) in relation to current and proposed vector management regimes. We estimate potential for arrival and survival of NIS and the change in these metrics anticipated under future requirements for ballast water treatment, recognizing that relative invasion risk is dependent on both the density of NIS carried by individual ships and the cumulative arrival of ships across time.

## Methods

### Estimating NIS arrival potential

**Fig 1:**
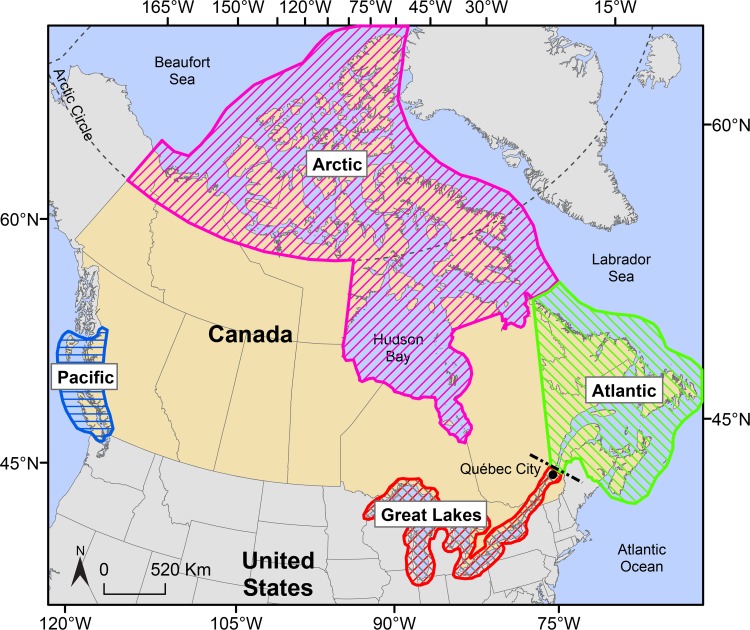
Geographic regions used to define shipping pathways and to quantify relative invasion risk among freshwater, brackish, and marine ecosystems (modified from Casas-Monroy et al. [27]).

A comprehensive database of the number and volume of ballast water discharges by merchant vessels (≥50m length with ballast capacity ≥8 m^3^) at ports in Canada and the Great Lakes was compiled using data extracted and cross-referenced from multiple Canadian and American government sources [27]. Because data were not available for the same year in all regions, comparative analyses were conducted using a 12-month time frame of recent data (2006, 2007 or 2008), representing an annual estimate of the arrival potential of NIS. Merchant vessels were grouped into geographic pathways based on their region(s) of operation (Table 1; Fig. 1). Biological data (density, diversity, biogeographic status of species in ballast water) were obtained from coordinated surveys conducted by the Canadian Aquatic Invasive Species Network (CAISN) and Fisheries and Oceans Canada over three years (2007 to 2009). Standardized protocols were used across the country to assess multiple taxonomic groups (diatoms, dinoflagellates and invertebrates) in ballast water sampled from different shipping pathways [17, 32–37], including unpublished data from Munawar, Fisheries and Oceans Canada. On average, 40 samples were collected for each region for zooplankton and 20 samples for phytoplankton (Table 1). For the East Coast, ballast water samples were collected in the ports of Sept-Îles, Port Cartier, Baie-Comeau (Quebec), St. John (New Brunswick), Canso, Halifax, Hantsport and Liverpool (Nova Scotia). For the West Coast, ships were sampled at the Port of Vancouver. In the Great Lakes, ships were sampled in Nanticoke, Hamilton (Ontario, Canada) and Toledo (Ohio, United States) [33]. Ships were selected on an opportunistic basis.

**Table 1:** Definitions of shipping pathways in Canada and the Great Lakes, with corresponding ballast water management requirements (modified from Casas-Monroy et al. [27]).

Pathway	Operation Region	Management Requirement	N
Arctic Coastal Domestic	GLSLR, Atlantic and Arctic regions	No exchange/flush required	13/NA
Eastern Coastal Domestic	GLSLR and Atlantic regions	No exchange/flush required	37/7
Lakers	GLSLR region and St. Lawrence Estuary (from Duluth to Sept Iles)	No exchange/flush required	87/6
Atlantic International Exempt	Atlantic region and coastal U.S. north of Cape Cod	No exchange/flush required	11/14
Pacific International Exempt	Pacific region, with last port-of-call in the coastal U.S. north of Cape Blanco	No exchange/flush required	17/23
Arctic International Transoceanic	GLSLR, Atlantic and Arctic regions and global ports outside Canada	Exchange/flush >200 nm offshore and >2000m depth prior to entering Canadian EEZ	23/22
GLSLR International Transoceanic	GLSLR region and global ports outside Canada		16/17
Atlantic International Coastal U.S.	Atlantic region and coastal U.S. (south of Cape Cod)	Exchange/flush >50 nm offshore and >500m depth prior to entering Canadian EEZ	23/23
Atlantic International Transoceanic	Atlantic region and global ports outside Canada	Exchange/flush >200 nm offshore and >2000m depth prior to entering Canadian EEZ	22/22
Pacific International Coastal U.S.	Pacific region and coastal U.S. (south of Cape Blanco)	Exchange/flush >50 nm offshore and >500m depth prior to entering Canadian EEZ	17/23
Pacific International Transoceanic	Pacific region and global ports outside Canada	Exchange/flush >200 nm offshore and >2000m depth prior to entering Canadian EEZ	23/24

GLSLR = Great Lakes-St. Lawrence River, EEZ = Exclusive Economic Zone. N = Number of samples for biological assessment of Zooplankton/Phytoplankton. NA = not assessed.

Arrival potential was assessed separately for zooplankton and phytoplankton at two timescales: the number of NIS discharged per event and the cumulative number of NIS discharged per year. Since plankton densities sampled from ballast water displayed high variation across ships within a given pathway, we conducted a simple resampling process to generate a mean arrival potential statistic for each timescale. First, we constructed a probability distribution of the observed density of NIS from tank samples within a given shipping pathway. Next, we randomly selected a density value from the probability distribution, which was multiplied by a randomly selected value of ship discharge volume. The result of this multiplication was one trial of the total number of NIS released to the recipient ecosystem associated with a single shipping event. This process was repeated across all of *n* discharges in a given year, and then across 1000 iterations. The mean value at each timescale represents the average number of NIS discharged (i.e., mean abundance of NIS released per event; mean released cumulatively within a given year). Five categorical bins ranging from lowest to highest arrival potential were created by determining the 20th, 40th, 60th, 80th, and 100^th^ percentiles from the entire distribution of values from the resampling process (e.g., for annual arrivals, percentiles were calculated from the 11,000 annual data points calculated for all pathways) [27]. We utilized the mean arrival values (which are influenced by right-tail skew in the distribution) derived during the Monte Carlo process to assign pathways into the percentile bins because discharges with very high NIS density, although rare, can be very important for invasion success [38].

### Estimating survival potential

Survival potential was estimated by comparing environmental similarity between paired source and recipient ports for each directional ballast water movement in the shipping database. We focused our analysis on salinity and climate (i.e., water temperature) because these are fundamental variables for the survival and reproduction of aquatic organisms (e.g., [39–41], and because including additional factors that are related to invasion risk for only a subset of all potential NIS can affect the sensitivity of the environmental similarity measure [42–44]. Global annual mean salinity was determined for each coastal source and recipient port using the online World Ocean Atlas database [45], while salinity of inland ports (e.g., Great Lakes ports) was obtained from Keller et al. [43]. Global ports were classified into salinity categories as follows: 0.0–5.0 parts per thousand (‰) as “fresh water”; 5.1–18.0 ‰ as “brackish”; 18.1 ‰ and higher as “marine”. To reflect the environmental effect of BWE, based on Canadian regulations requiring vessels to exchange ballast water resulting in a minimum final salinity of at least 30 ‰ [46], the source salinity was changed to 30 ‰ for all vessel transits that conducted BWE to reflect that the source of the ballast water was no longer from a coastal port, but from the ocean. A matrix approach was used to determine similarity of salinity between all source-recipient pairs [47]. The score has three metrics and ranged from “lowest” similarity of salinity for a port-pair with highly divergent salinities (e.g., freshwater—marine) to “highest” similarity if both ports had the same salinity classification (e.g., freshwater—freshwater). Following Spalding et al. [48] and Keller et al. [43], climate categories were assigned according to latitudinal port position as follows: Tropical (0°–20°N/S), Warm-Temperate (20°–40°N/S), Cold-Temperate (40°–60°N/S), and Polar (>60°N/S). Again a matrix approach was used to determine climate similarity between each source-recipient port-pair. The score has three metrics and ranged from “lowest” climate similarity for a port-pair with highly divergent climates (e.g., Polar-Tropical) to “highest” similarity if both ports were located in the same or adjacent climate category. The overall survival potential for each vessel transit was estimated based on the lowest score of either salinity or climate matching to reflect the influence of the most limiting environmental variable. The final survival potential for each shipping pathway was then assigned the level (highest, intermediate or lowest) having the greatest number of observations across voyages [27].

### Estimating relative invasion risk

The potentials for arrival and survival were combined into a final relative invasion risk for each pathway using a risk matrix (Table 2), modified from Chan et al. [44]. The risk was calculated using a 5 (arrivals) x 3 (survivals) matrix that reduces the final ratings to three levels. For example, given a lowest arrival potential and a highest survival potential, the overall introduction potential would be intermediate, because highest survival probabilities are offset by the small number of arriving individuals. Invasion risk was calculated separately for each pathway in each region, considering both annual and per-event arrivals and differential propagule pressures of zooplankton and phytoplankton.

**Table 2:** Matrix used to combine arrival potential and survival potential into final relative risk rankings.

		Arrival Potential				
		Lowest	Lower	Intermediate	Higher	Highest
**Survival potential**	**Highest**	Intermediate	Intermediate	Highest	Highest	Highest
	**Intermediate**	Lowest	Intermediate	Intermediate	Intermediate	Highest
	**Lowest**	Lowest	Lowest	Lowest	Intermediate	Intermediate

For example, given a lowest arrival potential and a highest survival potential, the overall introduction potential would be intermediate. At the opposite, given a highest rank of arrival potential and intermediate survival, the final relative risk invasion would be highest (modified from Casas-Monroy et al. [27]).

### Future invasion risk

Since Canada (and all other countries bound by the international convention, once fully ratified) will soon transition to a new ballast water management regime that is expected to enhance protection against ballast-mediated NIS, we repeated the process of estimating invasion risk using estimates of zooplankton/phytoplankton NIS densities expected after ballast water treatment. The anticipated NIS densities were estimated assuming the proportion of taxa (i.e., NIS vs. other taxa) will remain the same when the total abundance of all organisms is reduced to meet the D-2 standards. In addition, a salinity correction (30 ‰) was applied only for international transits (transoceanic or coastal) arriving to freshwater ports in line with requirements for “BWE plus treatment” on the Great Lakes [49–50].

## Results

### Baseline arrival potential

**Fig 2:**
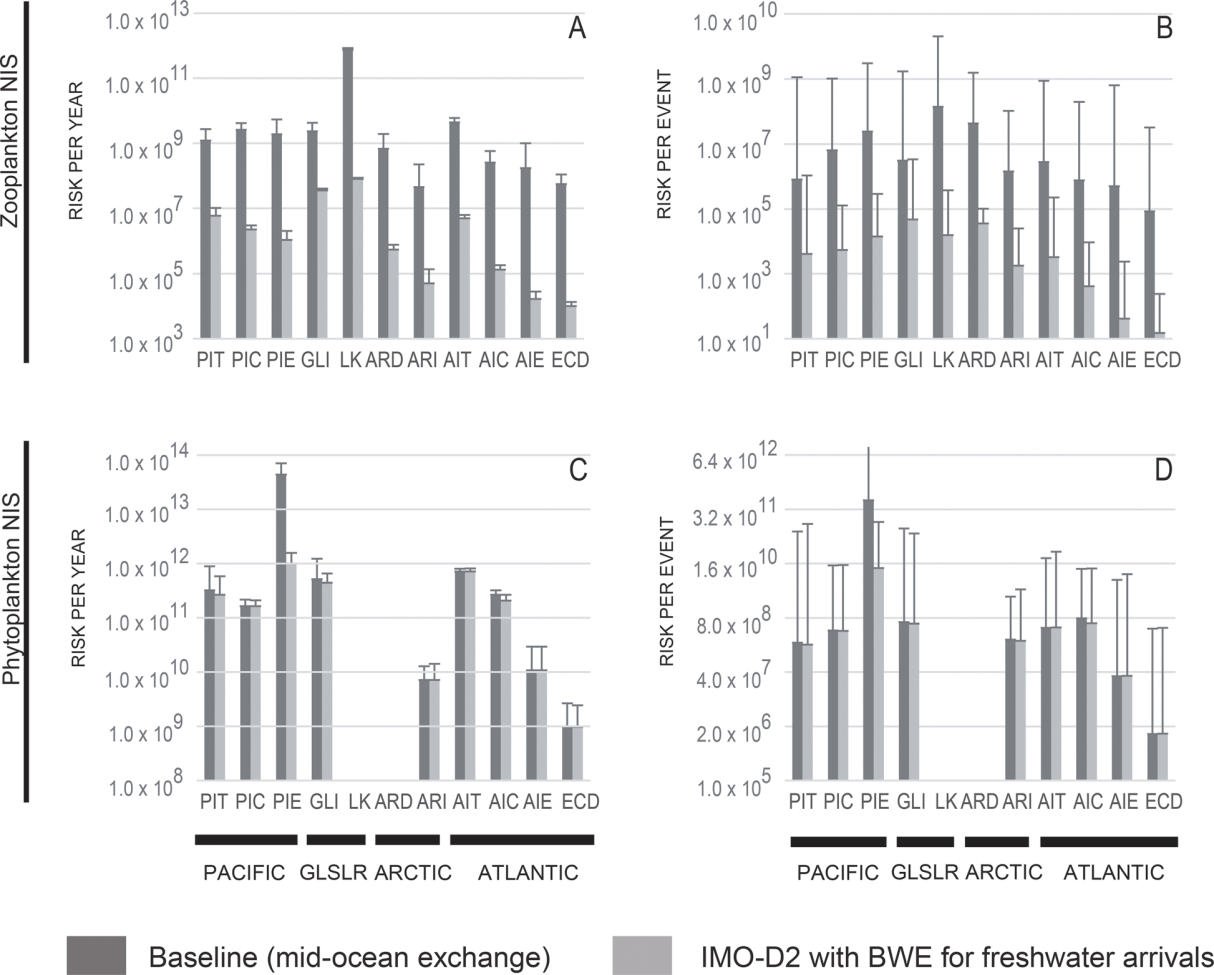
Estimated abundances of nonindigenous zooplankton (upper panels) and phytoplankton (lower panels) species transported in ballast water under current (dark gray) and future (light gray) management scenarios. Abundances were calculated on annual (left panels) and per event (right panels) timescales. PIT = Pacific International Transoceanic, PIC = Pacific International Coastal, PIE = Pacific International Exempt; GLSR = Great Lakes-St. Lawrence River International Transoceanic, LK = Lakers, ARD = Arctic Coastal Domestic, ARI = Arctic International Transoceanic, AIT = Atlantic International Transoceanic, AIC = Atlantic International Coastal, AIE = Atlantic International Exempt, ECD = Eastern Coastal Domestic.

Merchant vessels conducted roughly 11,000 ballast water discharge events at 309 Canadian and Great Lakes’ ports, discharging 116,159,585 m^3^ of ballast water during the twelve-month period assessed. Our results revealed a broad variance across pathways in terms of number of discharge events and annual volume of ballast water discharged (Table 3). Lakers and international transoceanic vessels are the most active pathways, in contrast with Arctic Coastal Domestic vessels. Hence, when factoring in biological data, the cumulative arrival potential for zooplankton NIS varied broadly, with the highest ranked pathways—Lakers and Atlantic International Transoceanic—transporting more than 8.33 x 10^11^ NIS per year, and the lowest ranked pathways—Arctic International Transoceanic—transporting less than 9.69 x 10^7^ NIS per year (Fig. 2A). Less variation was observed for the per event timescale, with the highest ranked pathways—Arctic Coastal Domestic and Lakers—transporting more than 8.24 x 10^9^ NIS per ship and the lowest ranked pathways—Eastern Coastal Domestic, Atlantic International Coastal U.S., and Pacific International Transoceanic—having less than 1.26 x 10^6^ NIS per ship (ranked as intermediate arrival potential) (Fig. 2B).

**Table 3:** Number of discharge events and annual volume of ballast water discharged by shipping pathways in Canada and the Great Lakes (modified from Casas-Monroy et al. [27]).

Pathway	Total Volume of Ballast Water Discharged	Number of discharge events
Arctic Coastal Domestic	78,125	16
Arctic International Transoceanic	197,589	30
Eastern Coastal Domestic	5,952,615	667
GLSLR International Transoceanic	2,914,206	759
Lakers	52,418,330	5227
Atlantic International Coastal U.S.	7,665,502	343
Atlantic International Exempt	5,652,994	357
Atlantic International Transoceanic	23,253,391	1530
Pacific International Coastal U.S.	2,324,543	415
Pacific International Exempt	592,089	79
Pacific International Transoceanic	15,110,203	1488

Cumulative annual arrival potential for phytoplankton NIS was also highly variable, with the highest ranked pathways—Atlantic International Transoceanic and Pacific International Exempt—introducing more than 7.02 x 10^13^ NIS per year, while the lowest ranked pathway—Lakers—introduces minimal NIS annually (Fig. 2C). No phytoplankton NIS were detected for Lakers in this study although the sample size was very small. The per-event arrival potential for phytoplankton NIS had an even larger range than annual levels, with highest ranked pathways—Arctic International Transoceanic, GLSLR International Transoceanic, Atlantic International Coastal U.S., Atlantic International Transoceanic, Pacific International Coastal U.S., Pacific International Exempt, and Pacific International Transoceanic—transporting more than 1.08 x 10^13^ NIS per ship, and the lowest ranked pathway—Lakers—again transporting negligible NIS (Fig. 2D).

### Baseline survival potential

**Fig 3:**
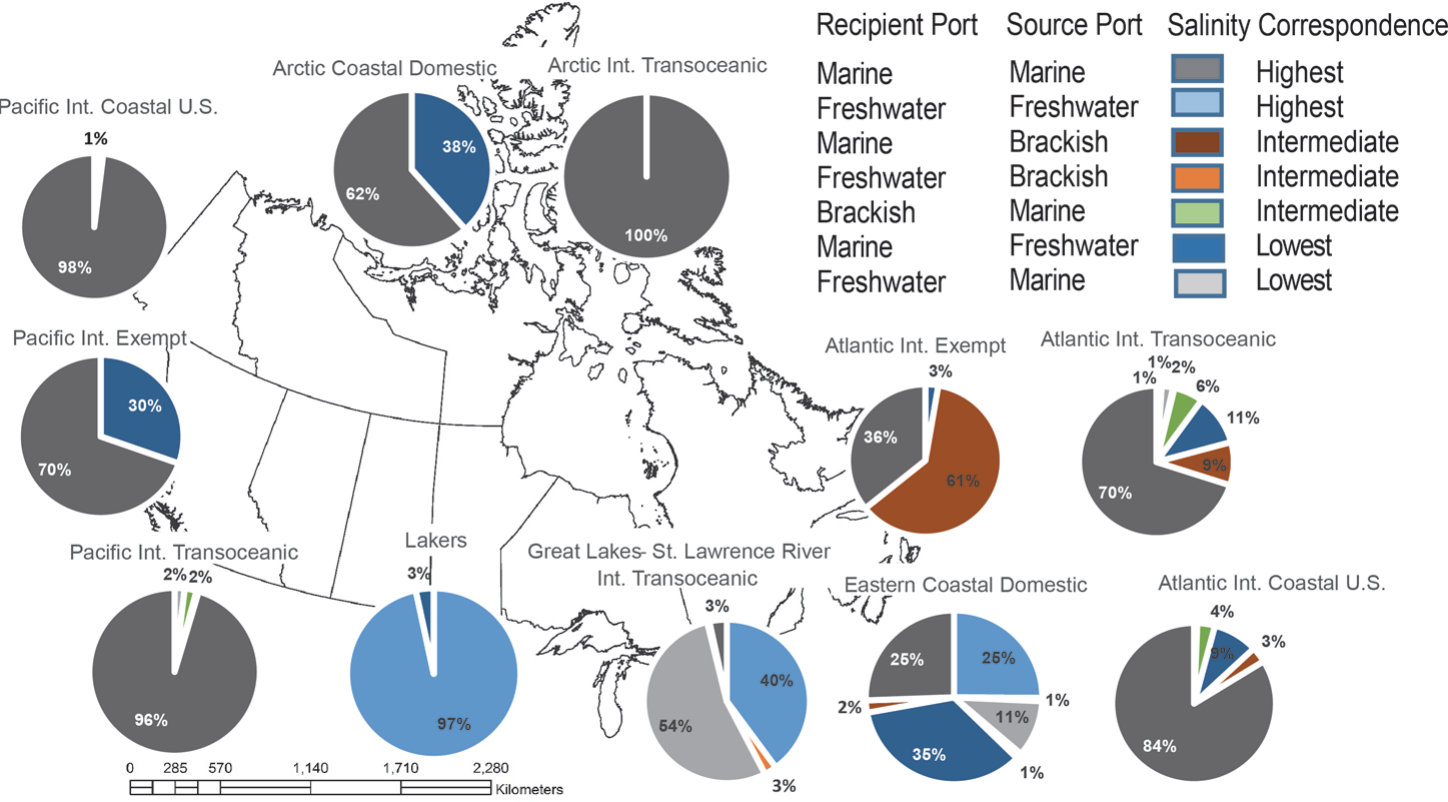
Estimated similarity of salinity between source and recipient locations of discharged ballast water under current requirements for ballast water exchange, summarized by pathway.

A total of 20,140 comparisons were conducted to evaluate environmental similarity between ballast water source-recipient port-pairs. Results indicate that 88% of vessel transits occurred between locations within the same salinity category. In total, seven pathways were ranked with highest salinity similarity, having more than 60% of vessel transits moving between locations of the same salinity category (Fig. 3). Only one pathway (Atlantic International Exempt) was ranked as intermediate with 61% of transits moving between locations where salinity was offset by one category. Finally, one pathway (GLSLR International Transoceanic) was ranked as lowest similarity with 54% of transits having dissimilar source-recipient salinities.

**Fig 4:**
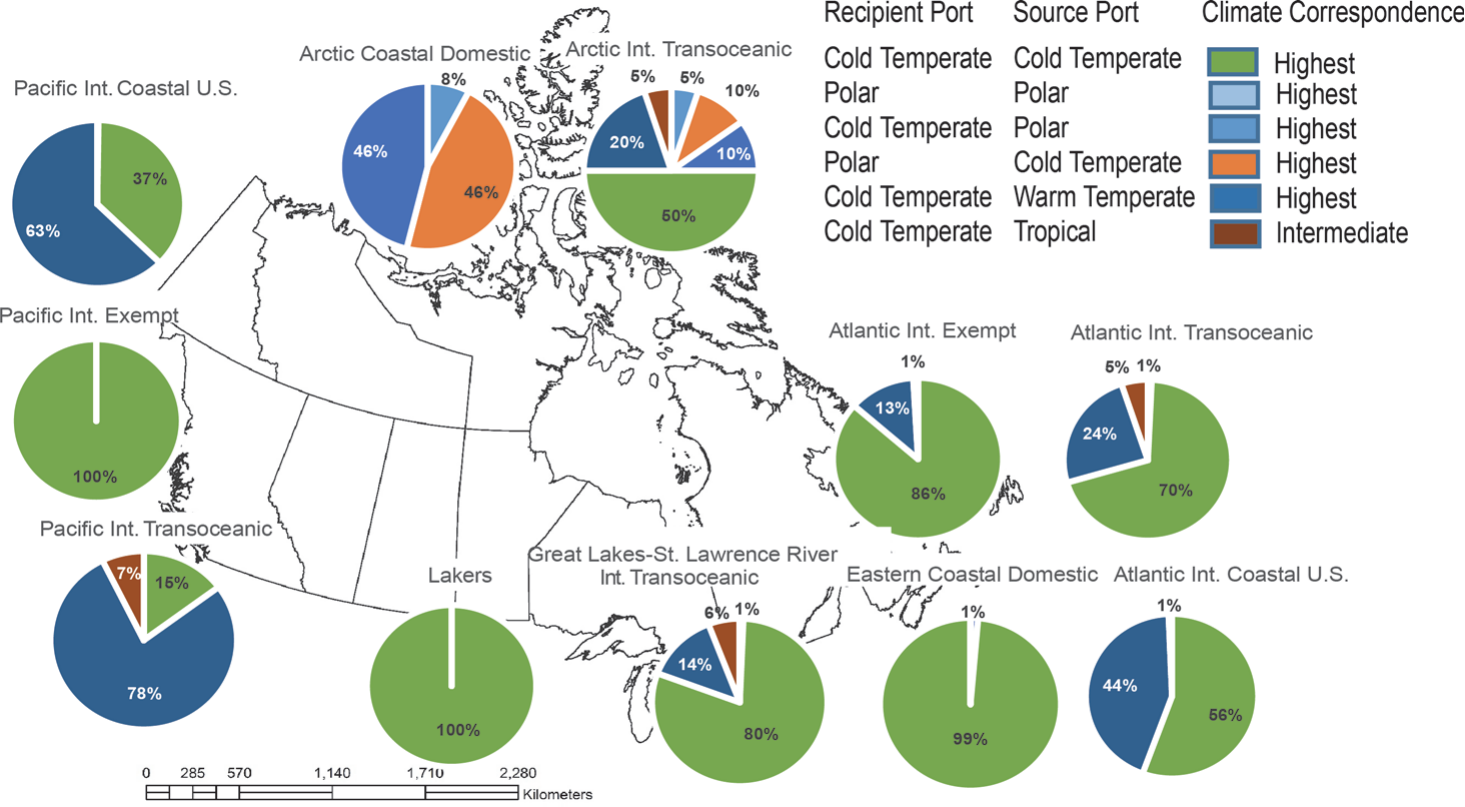
Estimated similarity of climate between source and recipient locations of discharged ballast water, summarized by pathway. Note that no combinations of Tropical-Polar transits were observed, so the lowest realized ranking is “Intermediate”. The same climate scenario was used for both current and future management scenarios.

Climate similarity results indicate that 87% of port-pair comparisons had highest similarity for climate. In total, all ten pathways were classified with highest climate similarity, having more than 60% of vessel transits moving between locations of the same climate category (Fig. 4). Our analysis showed that less than 10% of vessel transits occurred between locations having dissimilar climate.

The overall survival potential, based on the combination of salinity and climate similarity, was driven primarily by salinity rankings; the outcome for overall survival potential is therefore visually very similar to the results displayed in Fig. 3. Six pathways were ranked with highest survival potential, with 65% of transits moving between environmentally similar locations. Only one pathway (Atlantic International Exempt) was ranked with intermediate survival potential based on 60% of transits. Finally, two pathways (Arctic Coastal Domestic and GLSLR International Transoceanic) were ranked with lowest survival potential.

### Final relative invasion risk

**Fig 5:**
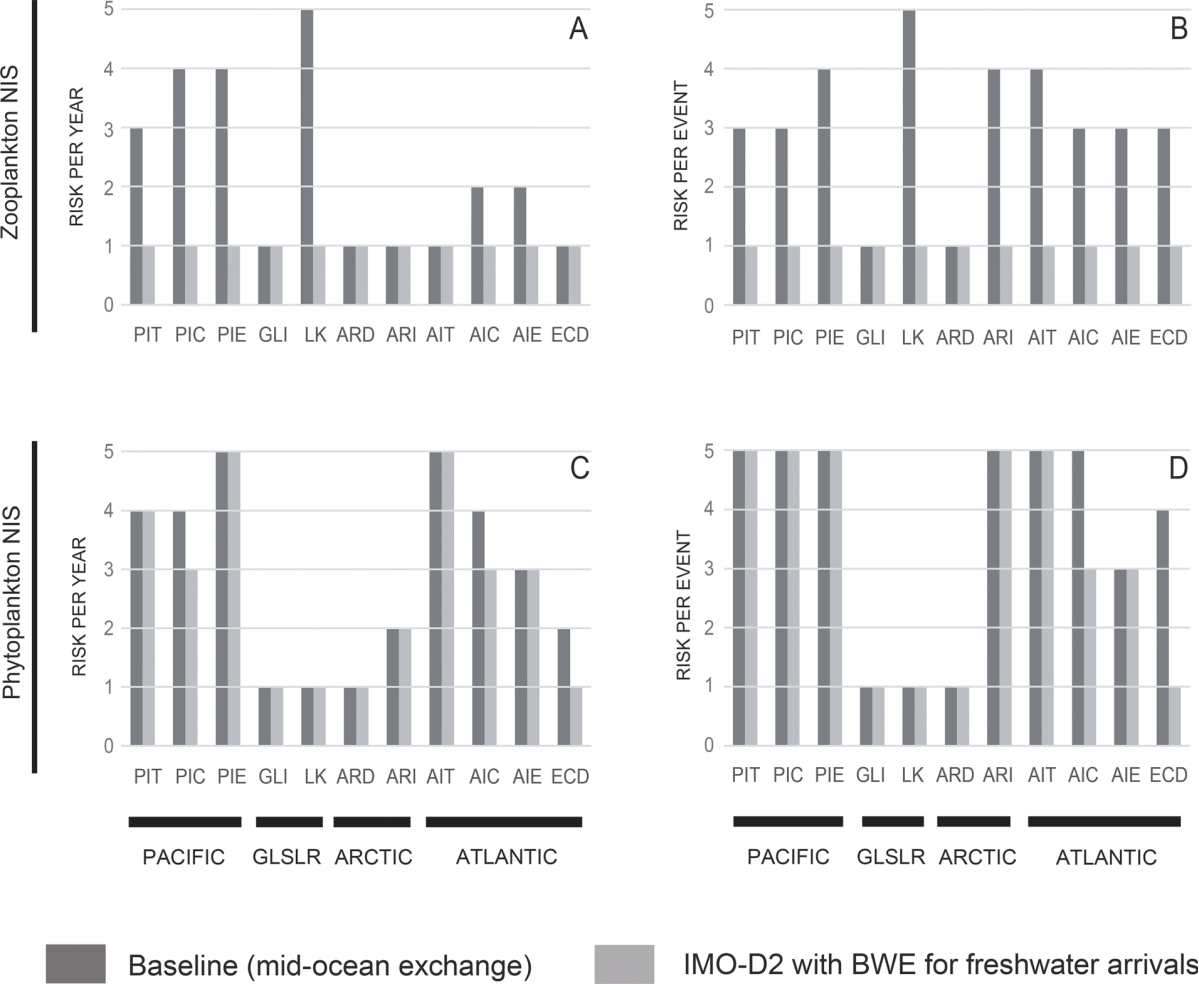
Final relative invasion risk estimated for nonindigenous zooplankton (upper panels) and phytoplankton (lower panels) species estimated under current (dark gray) and future (light gray) management scenarios. Invasion risk was calculated on annual (left panels) and per event (right panels) timescales. PIT = Pacific International Transoceanic, PIC = Pacific International Coastal, PIE = Pacific International Exempt; GLSR = Great Lakes Saint Lawrence International Transoceanic, LK = Lakers, ARD = Arctic Coastal Domestic, ARI = Arctic International Transoceanic, AIT = Atlantic International Transoceanic, AIC = Atlantic International Coastal, AIE = Atlantic International Exempt, ECD = Eastern Coastal Domestic. Risk scale bar denotes risk from lowest = 1 to highest = 5.

Our relative invasion risk assessment discriminated pathways into the following five groups: pathways presenting highest invasion risk for zooplankton NIS but lowest invasion risk for phytoplankton NIS on both temporal scales (Lakers), pathways posing higher invasion risk for zooplankton NIS at both temporal scales but highest for phytoplankton NIS (Pacific International Exempt) on an annual basis, pathways posing highest invasion risk for phytoplankton NIS at per event-scale (Pacific International Transoceanic, Pacific International Coastal U.S., Pacific International Exempt, Arctic International Transoceanic, Atlantic International Transoceanic, Atlantic International Coastal U.S.), pathways posing intermediate risk for zooplankton NIS at per event scale (Pacific International Transoceanic, Pacific International Coastal U.S., Atlantic International Coastal U.S., Atlantic International Exempt and Eastern Coastal Domestic), and finally, pathways posing lowest invasion risk for both taxonomic groups at both temporal scales (Great Lakes International Transoceanic and Arctic Coastal Domestic). The results of the final relative invasion risk assessment are summarized in Fig. 5 (A-D).

### Future risk with the IMO D-2 standards

Application of the IMO D-2 standards is expected to decrease abundances of zooplankton NIS for all pathways up to 99% in magnitude (Fig. 2A, B). In comparison to the current scenario, all pathways were ranked as lowest future arrival potential for zooplankton NIS on both annual and per event timescales. Although the ten pathways assessed already have mean densities of phytoplankton NIS below the future discharge standard, abundances of phytoplankton NIS may be expected to further decrease from 0.4% to 97% after application of IMO D-2 standards, since the variance in abundance will be reduced (Fig. 2C, 2D). In comparison to the current scenario, there are few changes in the ranking of marine international pathways for annual arrival potential (i.e., Atlantic International Coastal U.S. decreases its risk to intermediate), and no rankings change for per-event scenarios.

With the transition to treatment, rather than BWE, future survival potential based on similarity of salinity and climate is expected to decrease to intermediate for domestic pathways (i.e., Eastern Coastal Domestic) and one international pathway transiting between Canada and U.S. (i.e., Atlantic International Coastal U.S.) (data not shown). Again, the survival potential is driven by salinity of source-recipient locations. In the future scenario, where BWE is no longer required for vessels arriving to marine ports, environmental similarity decreases since many of the source ports are freshwater (see supplementary information, S1 Dataset, Raw data used for the relative invasion risk assessment).

Finally, Fig. 5A-5D) summarizes the final relative invasion risk assessment for the future scenario. Our results reveal that all pathways are expected to pose lowest invasion risk for zooplankton NIS, while future invasion risk for phytoplankton NIS ranges from lowest to highest. In comparison to the current scenario, the final relative invasion risk for zooplankton NIS decreased for seven pathways, but for phytoplankton NIS, decreased for only one pathway (Eastern Coastal Domestic).

## Discussion

The present relative risk framework incorporates NIS arrival and survival metrics to generate a landscape-scale portrait of the relative invasion risk across freshwater and marine ecosystems in Canada and the Great Lakes under current and future management regimes. Our findings show that current requirements for BWE reduce invasion risk to freshwater ecosystems, but are less effective in reducing risk to marine ecosystems because of greater environmental mismatch between source (oceanic) and recipient (freshwater) ecoregions. Future requirements for ballast water treatment are expected to reduce risk of zooplankton NIS introductions across ecosystem types but may be less effective in reducing risk of phytoplankton NIS. The proposed requirement for BWE plus treatment for vessels arriving to freshwater ports in the Great Lakes is expected to maintain very low survival potential of introduced organisms while systematically reducing propagule pressure; these expected benefits are supported by recent empirical tests [51].

This risk assessment indicates that international transoceanic ships show lowest invasion risk in the Great Lakes. Given that arrival potential by this pathway was similar or greater than that of all other pathways, our model confirms that the salinity mismatch induced by BWE is highly protective of this freshwater region [17]. In contrast, our model illustrates that domestic shipping (Lakers) can pose very high risk by facilitating secondary transfers of NIS, which can introduce novel genotypes in established populations, leading to greater impacts of NIS [52, 53] and patterns of dispersal that extend beyond a natural baseline.

The present model indicates the importance of short-sea coastal pathways on the Atlantic and Pacific coasts (currently exempt from BWE due to geographic proximity of ports), which appear to transport large numbers of NIS into Canadian marine waters at both timescales. Although, this pathway operates within a limited geographic extent, several source ports are considered primary introduction areas or “hotspots” (i.e., Great Bay, New Hampshire or Puget Sound, Washington) [54], with a high number of NIS that could be secondarily transported to Canadian ports. In both North America and Europe, recent studies have also pointed to coastal traffic as a critical vector introducing NIS [14, 15, 18, 55].

The model in the present study also reveals a high environmental similarity between source and recipient ports located along ocean coasts which are linked by pathways with global ports. This environmental similarity analysis is restricted to fundamental variables such as salinity and temperature in contrast with previous species-specific risk assessments. Using these two variables (salinity and climate as temperature proxy), we found that more than 80% of comparisons have highest environmental match. Over the coming century, sea surface warming at high latitudes is estimated to increase the level of environmental match between ports [56]. Consequently, an increase in shipping traffic connecting regions with high environmental match will expose regions of Canada to a larger number of NIS with high probability to establish populations, especially as a result of climate change.

This risk assessment suggests that shipping pathways managed by BWE do not necessarily exhibit lower risk than pathways without BWE; in part due to the number of transits—pathways with lower mean abundances of NIS can still pose a higher risk if there are many individual vessel transits. As noted in the introduction, the effect of BWE can be highly variable across individual ship transits [12, 15, 16, 18, 57]. In fact, in some studies BWE appears to have increased the risk of introduction potential of NIS, particularly for phytoplankton on the northwest Atlantic coast (e.g., 33, 58, 59]. Roy et al. [37] also reported that diversity of phytoplankton NIS was higher on ships that had undertaken BWE, with some species being of oceanic origin. Similarly, on the Pacific coast, Cordell et al. [12] reported that BWE had no significant influence on coastal zooplankton species, but increased the abundance of oceanic zooplankton species. Causes for BWE inefficiency may include structural limitations inside ballast tanks that restrict exchange of water, or offshore transport of coastal taxa [27]. Additionally, enforcement efforts (e.g. ballast water logs inspection, salinity measurements, salt flushing) are considerably lower for Canada’s marine coasts compared with the Great Lakes region [27].

Our future risk projections indicate that ballast water treatment at the level of the D-2 standards will dramatically reduce arrival potential for zooplankton NIS for all pathways in all regions but will have a lesser effect on arrival potential for phytoplankton NIS (reducing expected abundances of NIS for only five pathways). Hence, these results could be an indication that phytoplankton standards may not be as robust as zooplankton standards. According to the best available science at the time of drafting the D-2 standards, the limit for phytoplankton was set three orders of magnitude lower than the mean density observed in unmanaged ballast water while for zooplankton the limit was set two orders of magnitude lower [60]. The apparent dichotomy in our efficacy projections may be because our risk assessment evaluates the probability of NIS being introduced, whereas the D-2 standard does not discriminate between nonindigenous and more cosmopolitan taxa. In addition, recent advances in methods for the sampling and analysis of ballast water samples may have resulted in a shift of median plankton densities observed, compared to studies completed more than a decade ago. Evaluating the appropriateness of any particular ballast water discharge standard is a management exercise that involves risk tolerance, and is beyond the scope of this scientific risk assessment. The take home message of our analysis therefore should be that transition from BWE to treatment will result in improved protection against aquatic NIS across ecosystems and shipping pathways, with greater risk reduction expected for zooplankton as compared to phytoplankton.

The results presented in this research are based on recent shipping patterns and environmental metrics; any changes to one or both factors will lead to changes in relative invasion risk. Efforts to increase trade and shipping traffic would result in higher propagule arrival and could establish new connections with global source ports sharing high environmental similarity with recipient ports. Further, climate change scenarios predict both thermal and physical changes that could impact analyses of environmental similarity between source-recipient port-pairs. A reanalysis of environmental similarity between donor and recipient port-pairs, using environmental variables projected under climate change scenarios, may be useful to further refine predictions of future invasion risk [57].

Although biological invasions are highly stochastic, quantifying the arrival and survival of nonindigenous propagules across heterogeneous ecosystems represents a major step towards understanding the likelihood of invasion in relation to shipping networks, the relative efficacy of invasion management regimes, and seizing opportunities to reduce the ecological and economic implications of species invasion. Large-scale models assessing the invasion process can provide a general framework in order to better predict potential success of invading species [61] by including stochastic events [62]. [29]

## Supporting Information

S1 Dataset Raw data used for the relative invasion risk assessment. The file is divided in three sections: 1) ballast water discharge volumes for all the ships arriving to the Canadian regions using a 12-month time frame of recent data (2006, 2007 and 2008) representing an annual estimate of the arrival potential NIS. 2) Biological data obtained from coordinated surveys conducted by the Canadian Aquatic Invasive Species Network (CAISN) and Fisheries and Oceans Canada (DFO) over three years (2007 to 2009). 3) List of source-recipient port-pair and results of the overall survival potential, based on the combination of salinity and climate similarity.
(XLSX)
